# Exendin-4 as a multi-target therapy for Fetal Inflammatory Response Syndrome-induced brain injury

**DOI:** 10.3389/fneur.2026.1848474

**Published:** 2026-08-13

**Authors:** Shan Li, Cheng Cai, Gaowa Arigong

**Affiliations:** 1 Department of Neonatology, School of Medicine, Shanghai Children’s Hospital, Shanghai Jiao Tong University, Shanghai, China; 2 Neonatology Division, Inner Mongolia Maternity and Child HealthCare Hospital, Hohhot, Inner Mongolia, China

**Keywords:** cerebral white matter injury, Exendin-4, Fetal Inflammatory Response Syndrome, glucagon-like peptide-1 receptor agonist, network therapy, neuroinflammation, neuroprotection, precision medicine

## Abstract

Fetal Inflammatory Response Syndrome (FIRS) is a leading cause of neonatal brain injury for which no specific treatment currently exists. This review proposes that Exendin-4, a glucagon-like peptide-1 receptor agonist, is a promising “network therapy” due to its multi-target actions against the interconnected pathologies of FIRS. We synthesize evidence that Exendin-4 can concurrently mitigate neuroinflammation, protect oligodendrocytes, stabilize the blood–brain barrier, and attenuate excitotoxicity. Furthermore, we explore emerging mechanisms such as microglial metabolic reprogramming and enhanced mitophagy. Finally, we critically evaluate the translational pathway, highlighting challenges in drug delivery and the therapeutic window, and outline a precision medicine framework for its clinical development. This work positions Exendin-4 as a key candidate for overcoming the therapeutic gap in FIRS-associated neuroprotection.

## 1 FIRS: an unmet clinical need and the rationale for a network therapy paradigm

Fetal Inflammatory Response Syndrome (FIRS) is defined as a state of systemic fetal immune activation precipitated by intrauterine infection, in which the diagnostic gold standard is a significant elevation of interleukin-6 (IL-6) in umbilical venous blood (1, 2). The epidemiological data are alarming: intrauterine infection and inflammation are strongly implicated in up to 40% of all spontaneous very preterm births (occurring before 28 weeks of gestation), thereby exposing the immensely vulnerable and explosively developing fetal brain to a devastating and unchecked “inflammatory storm” (3). The neuropathological consequences are profound and enduring. FIRS is recognized as the predominant driver of diffuse white matter injury (WMI), a condition defined by periventricular leukomalacia (PVL) and characterized by extensive and selective loss of oligodendrocyte precursors (OPCs) accompanied by a secondary catastrophic failure of the myelination process (4). This neurostructural devastation directly translates into a dishearteningly high incidence of lifelong neurodevelopmental disorders (NDDs), encompassing spastic diplegic cerebral palsy, significant cognitive dysfunction, learning disabilities, attention-deficit/hyperactivity disorder (ADHD), and visual processing abnormalities (5, 6). The current therapeutic arsenal is notably inadequate for this specific pathology. Therapeutic hypothermia, while a cornerstone for hypoxic–ischemic encephalopathy, demonstrates no efficacy for FIRS-related WMI and lacks a plausible mechanistic basis for application. Furthermore, no specific pharmacological interventions exist that are designed to preemptively or therapeutically address the unique inflammatory cascade of FIRS (7). A critical and paradoxical observation underscores the inadequacy of current approaches: while advances in neonatal intensive care have markedly improved survival rates for very preterm infants, the concomitant incidence of NDDs has frustratingly failed to decline, highlighting a fundamental and persistent deficiency in specific, effective neuroprotective strategies tailored to FIRS-associated WMI (7). Consequently, the development of novel, mechanistically targeted strategies capable of traversing both the placental and blood–brain barriers, precisely modulating the dysregulated neuroimmune homeostasis, and actively supporting white matter repair constitutes a paramount and urgent medical mission of our time.

This review therefore aims to introduce and rigorously defend a central thesis: The pathological cascade initiated by FIRS is not a linear sequence of events but rather constitutes a self-reinforcing, dynamic “tumor-associated network” of inflammatory, cellular, and vascular injuries, each component exacerbating the others. The networked nature of this injury necessitates a corresponding “network therapy” paradigm—an intervention capable of multi-target, synergistic engagement. We present a comprehensive argument that Exendin-4, a glucagon-like peptide-1 receptor agonist with a demonstrated pleiotropic pharmacodynamic profile, is a promising candidate for this role. However, we further contend that its successful translation from bench to bedside hinges critically on a fundamental paradigm shift from the historical concept of broad, non-specific neuroprotection to mechanism-based, biomarker-informed, precision approaches. We will critically evaluate the cumulative evidence supporting this claim, delineate the formidable translational challenges with intellectual honesty, and propose a strategic and innovative roadmap for future research.

## 2 Method

We searched the following databases: PubMed, Web of Science, Scopus, and Google Scholar. The search terms included combinations of “Fetal Inflammatory Response Syndrome,” “FIRS,” “Exendin-4,” “GLP-1 receptor agonist,” “neuroinflammation,” “oligodendrocyte,” “blood–brain barrier,” “excitotoxicity,” “microglial metabolic reprogramming” and “mitophagy.” The inclusion criteria were: (1) original research articles, reviews, and meta-analyses published in English; (2) studies focusing on FIRS, neuroinflammation, or related mechanisms; (3) studies investigating Exendin-4 or GLP-1 receptor agonists in neuroprotection. Exclusion criteria were: (1) non-English publications; (2) studies not directly relevant to FIRS or Exendin-4 mechanisms; (3) conference abstracts without full text. The time period covered was from January 2000 to October 2023, with a focus on recent advances.

## 3 Core pathophysiological mechanisms of FIRS-induced brain dysplasia: a deconstruction of the “malignant network”

A critical and nuanced synthesis of the contemporary literature demonstrates that FIRS-induced brain injury is not a linear process, but rather a dynamic, multi-stage, and self-amplifying “malignant network” (2, 7). Its core pathophysiological mechanisms can be categorized into four deeply intertwined and mutually reinforcing dimensions, a complexity that itself underscores the need for a multi-target therapeutic strategy such as Exendin-4.

### 3.1 Dysregulation of the neuroimmune axis: the central role of microglial polarization imbalance

The developing central nervous system possesses a unique and delicately balanced immune ecosystem, wherein microglia, the resident innates immune cells, play indispensable and multifaceted roles in neural circuit shaping, synaptic pruning, and myelin maintenance (8). In the context of FIRS, peripherally generated inflammatory signals, including pathogen-associated molecular patterns (PAMPs) and damage-associated molecular patterns (DAMPs), successfully breach fetal immune defenses. PAMPs, such as lipopolysaccharide (LPS) and bacterial CpG DNA, are primarily derived from intra-amniotic infection, while DAMPs, including high-mobility group box 1 (HMGB1) and extracellular ATP, are released from the compromised placenta and damaged fetal tissues (representative molecules are detailed in Table 1). These signals propagate to the fetal brain through multiple routes: (i) direct translocation across a compromised placenta and an immature blood–brain barrier; (ii) indirect signaling via the activation of immune-competent cells in pericentral brain regions such as the choroid plexus; and (iii) potentially, through vagus nerve-mediated neurohumoral pathways. Upon reaching the CNS, these molecules activate microglia via pattern recognition receptors (e.g., TLRs, RAGE), initiating a neuroinflammatory cascade that disrupts normal brain development (9). The aforementioned pathway has been summarized in Table 1.

**Table 1:** Key PAMP and DAMP molecules implicated in Fetal Inflammatory Response Syndrome (FIRS) and their potential pathways to the fetal brain.

Category	Representative molecule	Primary source	Key receptor(s)	Example References
PAMPs	Lipopolysaccharide (LPS)	Intra-amniotic Gram-negative bacteria (e.g., *E. coli*)	TLR4	(68)
	Lipoteichoic acid (LTA)	Intra-amniotic Gram-positive bacteria (e.g., Group B Streptococcus)	TLR2	(69)
	Bacterial CpG DNA	Bacterial genomic DNA	TLR9	(70)
	Peptidoglycan (PGN)	Bacterial cell wall	TLR2 / NOD2	(71)
DAMPs	High-mobility group box 1 (HMGB1)	Necrotic/apoptotic placental trophoblasts; activated immune cells	TLR2/4, RAGE	(72)
	Extracellular ATP	Damaged or stressed cells	P2X7 receptor	(73)
	Heat shock proteins (HSP60, HSP70)	Stressed or necrotic cells	TLR2/4	(74)
	S100 proteins (S100A8/A9)	Activated neutrophils/monocytes; damaged tissue		(74)

This systemic inflammatory insult triggers a profound phenotypic shift of microglia from a surveillant, homeostatic state, characterized by neuroprotective M2-like properties, into a highly activated, neurotoxic “classical activation” (M1) phenotype (10, 11).

These M1-polarized microglia act as “inflammatory amplifiers,” releasing substantial quantities of pro-inflammatory cytokines (most notably IL-1β, TNF-*α*, and IL-6), chemokines (e.g., MCP-1), reactive oxygen and nitrogen species (ROS/RNS), and excitatory amino acids like glutamate (12). Among these, TNF-α and IL-1β have been shown to directly induce cell cycle arrest, impair differentiation, and trigger caspase-3-dependent apoptosis in oligodendrocyte precursors (OPCs) (12). Compounding this injury, activated astrocytes undergo reactive astrogliosis, further exacerbating synaptic over-pruning through the release of complement components (e.g., C1q, C3) and potentially forming glial scars that act as physical and chemical barriers to axonal regeneration and myelin repair (13). The recently elucidated concept of “trained immunity” or “innate immune memory” introduces a further layer of complexity. It suggests that a transient inflammatory stimulus like FIRS may induce persistent metabolic and epigenetic reprogramming in microglia, priming them for exaggerated and potentially damaging inflammatory responses to subsequent minor challenges throughout life; this process represents a novel mechanism by which FIRS could exert a lasting negative impact on long-term brain health and resilience (14). This pervasive and persistent state of immune dysregulation thus represents a critical, imperative target for any effective intervention.

In the context of FIRS, it is essential to first distinguish between resident microglia in the brain and peripherally infiltrating macrophages that may be pathological. Microglia originate from yolk sac progenitors and self-renew throughout life within the brain, constituting the principal immune effector network in the developing brain parenchyma. These activated M1-type microglia effectively function as “inflammatory amplifiers,” secreting substantial quantities of pro-inflammatory cytokines (most notably IL-1β, TNF-*α*, and IL-6), chemokines (such as MCP-1), reactive oxygen/nitrogen species (ROS/RNS), and excitatory amino acids such as glutamate (15). Concurrently, microglia-derived ROS/RNS directly damage myelin sheaths. This combined assault severely disrupts myelination, a cornerstone of the functional development of neural circuits. These inflammatory signals widely compromise neuronal health and connectivity by directly threatening neuronal integrity. Excessive glutamate from activated microglia, along with TNF-*α*, can lead to excitotoxic neuronal death. Furthermore, prolonged exposure to cytokines such as IL-1β alters synaptic plasticity and weakens neurotrophic support. Compounding these effects, activated astrocytes undergo reactive gliosis, further exacerbating synaptic over-pruning by releasing complement components (e.g., C1q, C3) and potentially forming glial scars that act as physical and chemical barriers to axonal regeneration and myelin repair (16). The recently elucidated concept of “trained immunity” or “innate immune memory” introduces a deeper layer of complexity. This suggests that a transient inflammatory stimulus such as FIRS may induce persistent metabolic and epigenetic reprogramming in microglia, thereby priming them to mount excessive and harmful inflammatory responses to mild challenges later in life (17). This represents a novel mechanism through which FIRS may exert a lasting detrimental influence on brain health and resilience. Therefore, this widespread and persistent state of immune dysregulation is a key target that any effective intervention must address.

### 3.2 Oligodendrocyte spectrum damage: the cellular and molecular basis of impaired myelination

Within the context of FIRS, the oligodendrocyte (OL) lineage exhibits a marked, stage-specific susceptibility to the hostile inflammatory milieu, which is characteristically dominated by microglial-derived cytokines such as TNF-*α* and IL-1β. Late OPCs and immature oligodendrocytes exhibit heightened vulnerability to this FIRS-associated cytokine storm, which induces apoptosis and profoundly derails their tightly regulated differentiation program (18). The molecular underpinnings of this inflammation-induced arrest are increasingly understood; for instance, inflammatory signals drive persistent activation of the Wnt/β-catenin signaling pathway, effectively imposing a developmental “lock” that maintains OPCs in a proliferative state while inhibiting terminal differentiation into mature, myelin-producing oligodendrocytes (19). Concurrently, the same inflammatory environment significantly disrupts neurotrophic factor signaling pathways critical for OL survival and maturation, such as the BDNF/TrkB axis (20). Furthermore, FIRS-induced microglial activation and cytokine release contribute to axonal damage and dysfunction, thereby depriving developing OLs of essential, contact-dependent axonal signals (e.g., Neuregulin-1) required for final maturation and myelination initiation (21). The cumulative effect of these FIRS-mediated mechanisms is a severe deficiency in myelin, characterized by structurally abnormal, thin sheaths with shortened internodes, which directly impairs nerve conduction velocity and desynchronizes neural networks. This microstructural disarray, rooted in the specific inflammatory insult of FIRS, forms the fundamental substrate for the cognitive, motor, and sensory deficits observed clinically.

### 3.3 Blood–brain barrier disruption: a critical hub in the vicious cycle of inflammation

The developing blood–brain barrier (BBB) is structurally and functionally immature, rendering it highly vulnerable to injury during FIRS. Key pro-inflammatory cytokines elevated in FIRS, especially TNF-*α*, induce the downregulation and disruption of critical tight junction proteins (e.g., ZO-1, occludin) and simultaneously upregulate endothelial adhesion molecules (e.g., ICAM-1) (22). This FIRS-mediated pathophysiological remodeling results in two primary consequences: (i) a significant increase in BBB permeability, permitting a greater influx of systemic inflammatory mediators (which may include PAMPs from intra-amniotic infection), peripheral immune cells, and serum proteins into the brain parenchyma, thereby further amplifying the local neuroinflammatory response; and (ii) a functional decoupling of the neurovascular unit (NVU), impairing cerebral blood flow regulation and potentially precipitating secondary ischemic injury within the vulnerable white matter (23). Therefore, BBB disruption in FIRS is not a passive consequence but an active and critical driver that perpetuates the “cancer network,” thereby positioning its stabilization as a pivotal therapeutic strategy essential for arresting the FIRS cascade.

### 3.4 Excitotoxicity, oxidative stress, and mitochondrial dysfunction: converging downstream executioner pathways

The FIRS-induced inflammatory microenvironment directly perturbs cerebral glutamate homeostasis. This process is mediated by microglial activation and reactive astrogliosis, thereby inhibiting astrocytic glutamate reuptake (via down-regulation of EAAT2) and promoting excessive glutamate release (23). The resultant excitotoxic milieu in the FIRS-affected brain induces hyperactivation of glutamate receptors on neurons and oligodendrocytes, thereby precipitating a toxic intracellular calcium influx. This calcium overload, a hallmark of FIRS-associated injury, initiates a cascade characterized by mitochondrial membrane potential collapse, energetic failure, exacerbated mitochondrial ROS generation, and the activation of calcium-dependent proteases, culminating in cell death pathways (24). Mitochondrial dysfunction, at the nexus of energy production and redox balance, serves as the “final common pathway” through which FIRS-related inflammatory and excitotoxic insults converge to mediate cell death. Targeting this convergence point constitutes a promising therapeutic strategy for bolstering cellular resilience in the FIRS-compromised brain.

## 4 GLP-1RAs and Exendin-4: a paradigm shift from glucose-lowering agents to pleiotropic CNS-active neuroprotectors

**Figure 1:**
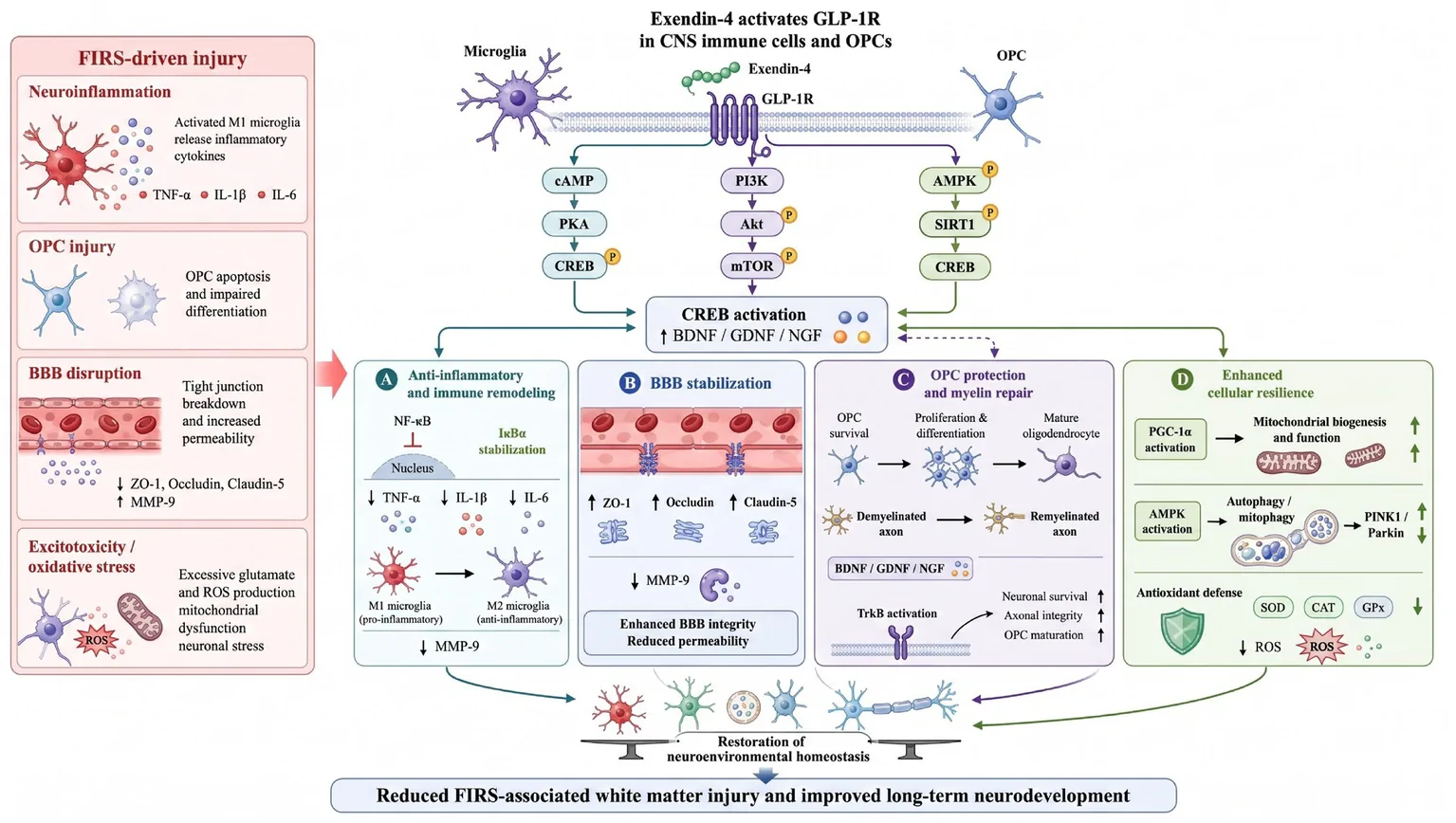
Details the mechanisms by which FIRS causes injury and the four pathways through which Exendin-4 activates GLP-1R in CNS immune cells and OPCs, thereby elucidating the alleviation of FIRS-related white matter damage and improves long-term neurodevelopment.

The scientific and clinical understanding of glucagon-like peptide-1 receptor agonists (GLP-1RAs) has undergone a profound transformation over the past decade. While the native GLP-1 hormone, secreted primarily by intestinal L-cells, has an extremely short plasma half-life (approximately 1–2 min) due to rapid degradation by the dipeptidyl peptidase-4 (DPP-4) enzyme, Exendin-4 offers a distinct advantage. This 39-amino acid peptide originally isolated from the saliva of the Gila monster (*Heloderma suspectum*), functions as a high-affinity, long-acting agonist of the GLP-1 receptor and is naturally resistant to DPP-4-mediated degradation, resulting in a significantly extended half-life of about 2.4 h and making it an potential tool for scientific investigation and a valuable drug lead compound (25, 26). The synthetic analogue of Exendin-4, exenatide, has been approved for the clinical management of type 2 diabetes mellitus, as illustrated in the schematic diagram in Figure 1.

The therapeutic potential of Exendin-4 in FIRS is predicated on its mechanism of action as a high-affinity, long-acting agonist of the glucagon-like peptide-1 receptor (GLP-1R) (27). Critically, GLP-1R is expressed by key cellular targets within the fetal neuro-immune plexus, including microglia and oligodendrocyte precursor cells (OPCs) (28, 29). Upon binding, Exendin-4 mediates a dual-pathway mechanism: in microglia, GLP-1R activation suppresses NF-κB signaling, thereby inhibiting the release of pro-inflammatory cytokines and reactive oxygen species, thereby attenuating the primary inflammatory response. Concurrently, direct GLP-1R stimulation of OPCs activates PI3K/Akt and CREB-dependent survival pathways, which inhibit apoptosis and promote differentiation into myelinating oligodendrocytes (30). This coordinated modulation of both the inflammatory milieu and the vulnerable OL lineage exemplifies a comprehensive network therapy approach (31), simultaneously disrupting the damaging “neuro-immune complex” and bolstering endogenous repair mechanisms to restore myelination and neural circuit function. The mechanisms underlying the GLP-1R, cell type, and downstream pathway interactions are summarized in Table 2.

**Table 2:** Overview of the GLP-1R mechanisms linking receptor activation, cell types, and downstream signaling pathways.

Target cells	Receptor	Key signaling pathways	Protective effect	Pathology of direct confrontation	References
Microglia	GLP-1R	cAMP/PKA → inhibition of NF-κB; AMPK/SIRT1 → M2-type metabolic reprogramming	Inhibits the release of TNF-α, IL-1β and IL-6; promotes M1-to-M2 polarization; reduces the density of Iba1 + cells	Neuroinflammatory storm, excitotoxicity	(30–33)
OPCs/oligodendrocytes	GLP-1R	PI3K/Akt/mTOR; CREB/BDNF; Nrf2/HO-1	Reduces apoptosis and promotes proliferation, differentiation and MBP expression; accelerates myelin regeneration; protects against oxidative damage	OPC damage, impaired myelination	(34–37)
Cerebral vascular endothelial cells	GLP-1R	Inhibition of NF-κB/MMP-9; upregulation of cAMP/PKA → stabilization of junctional proteins	Increases ZO-1, occludin and claudin-5; reduces IgG/Evans blue leakage;	BBB disruption	(38)
			maintains BBB integrity		
Neurons/synapses	GLP-1R	Modulation of NMDA receptor function; PGC-1α → mitochondrial biogenesis; enhancement of autophagy/mitochondrial autophagy	Alleviates glutamate excitotoxicity; restores calcium homeostasis; increases ATP production; eliminates damaged mitochondria	Excitotoxicity, oxidative stress, mitochondrial dysfunction	(39–41)
(Indirect) gut microbiota	–	Increase the abundance of SCFA-producing bacteria (indirect effect)	It may enhance BBB function and regulate microglia via the gut-brain axis	Chronic neuroinflammation	(42)

### 4.1 Neuroprotective mechanisms of Exendin-4 and GLP-1RAs: a multi-target synergistic network

A substantial and growing body of preclinical evidence now strongly supports Exendin-4, and the GLP-1RA class more broadly, as potent neuroprotective agents, exerting effects largely independent of their glucose-lowering actions. The protective mechanisms mediated by these agents form a sophisticated, multimodal, and synergistic network that is uniquely suited to counteracting the FIRS “malignant network.”

Potent and Broad-Spectrum Anti-inflammatory Actions: In various CNS disease models, Exendin-4 has been consistently shown to inhibit the activation of both microglia and astrocytes, reduce the expression and release of M1 polarization markers (e.g., iNOS, CD86) and key pro-inflammatory cytokines (TNF-*α*, IL-1β, IL-6), while concurrently promoting a phenotypic shift toward the anti-inflammatory and pro-repair M2 state, as evidenced by increased expression of Arg-1, CD206, and IL-10, thereby actively remodeling the neuroimmune microenvironment into a state conducive to recovery and repair (32).

Robust Anti-apoptotic and Pro-survival Signaling: Upon activation of the GLP-1 receptor, Exendin-4 elevates intracellular cyclic AMP (cAMP) levels via Gs protein coupling, which in turn activates protein kinase A (PKA) and its downstream transcription factor, cAMP response element-binding protein (CREB). In parallel, it potently activates the pro-survival PI3K/Akt signaling pathway, while inhibiting several pro-apoptotic pathways, including c-Jun N-terminal kinase (JNK) and the nuclear factor kappa-light-chain-enhancer of activated B cells (NF-κB). The concerted action of these signaling events results in the transcriptional downregulation of pro-apoptotic proteins (e.g., Bax, Bad) and upregulation of anti-apoptotic proteins (e.g., Bcl-2, Bcl-xL), and the subsequent inhibition of effector caspase-3, thereby preserving cellular integrity and viability (28).

Comprehensive Antioxidative Stress Capabilities: Exendin-4 enhances cellular intrinsic antioxidant defense system, characterized by increased activity of key enzymes such as superoxide dismutase (SOD), catalase (CAT), and glutathione peroxidase (GPx). Furthermore, it directly inhibits the activity of major ROS-generating enzymes, particularly NADPH oxidase, thereby reducing the burden of excess free radicals and mitigating oxidative damage to cellular macromolecules (33, 34).

Promotion of Essential Neurotrophic Support: GLP-1 receptor activation significantly upregulates the expression of a range of critical neurotrophic factors, including brain-derived neurotrophic factor (BDNF), glial cell line-derived neurotrophic factor (GDNF), and nerve growth factor (NGF). These factors function via both autocrine and paracrine mechanisms to promote the survival, differentiation, synaptic plasticity, and overall metabolic health of neurons and oligodendrocytes (35).

Improvement of Mitochondrial Function and Induction of Biogenesis: Exendin-4 stabilizes the mitochondrial membrane potential, enhances the efficiency of the oxidative phosphorylation system, and consequently increases cellular ATP production. Notably, it actively promotes the generation of new, healthy mitochondria through the activation of the PGC-1α (peroxisome proliferator-activated receptor gamma coactivator 1-alpha) signaling axis, thereby providing the essential energy supply required for the recovery and proper function of injured neural cells (36).

Regarding the protection of the blood–brain barrier (BBB) and neurovascular unit, a growing body of evidence from models of cerebral ischemia and diabetic encephalopathy demonstrates that Exendin-4 treatment effectively attenuates BBB disruption and hyperpermeability. These multifaceted mechanisms include the upregulation of tight junction protein expression, the direct inhibition of matrix metalloproteinase-9 (MMP-9) activity (a key enzyme responsible for degrading the extracellular matrix and junctional proteins), and the attenuation of perivascular inflammation; together, these actions preserve the structural and functional integrity of the neurovascular unit (37).

A Critical Translational Bottleneck and Imperative for Innovation: Despite this exceptionally promising and multifaceted pharmacodynamic profile, a significant limitation remains the limited permeability of Exendin-4 across the intact blood–brain barrier following systemic administration in adults. This poses a critical pharmacokinetic hurdle for its clinical translation in neurological disorders, including FIRS. Overcoming this bottleneck necessitates the development of innovative CNS-targeted drug delivery strategies, a topic that will be explored in depth in the final section of this review.

## 5 Potential mechanisms of Exendin-4 against FIRS brain damage: a detailed and evidence-based mechanistic extrapolation

While the direct application of Exendin-4 in well-characterized FIRS models remains an area of active and necessary investigation, we can develop a mechanistically grounded hypothesis regarding its considerable potential by critically integrating and interpreting findings from a wide spectrum of related neonatal brain injury models (e.g., hypoxic–ischemic encephalopathy, other intrauterine inflammation paradigms) and established models of neuroinflammatory and neurodegenerative diseases. This approach enables the formulation of a compelling and sophisticated rationale for its efficacy. Recent transcriptome-wide studies have begun to elucidate the complex molecular networks underlying FIRS. RNA-seq and microarray analyses of brain tissue from preclinical models of intrauterine inflammation consistently reveal a coordinated transcriptional reprogramming, with significant upregulation of pathways associated with innate immunity and pro-inflammatory signaling (IL-6, TNF-*α*, TLR signaling) and concurrent suppression of gene modules essential for oligodendrocyte maturation and myelination (MBP, PLP1) (38, 39). These genomic signatures provide direct evidence that FIRS-induced inflammation profoundly disrupts both neuroimmune and neurodevelopmental programs, establishing a complex pathogenic network. Table 3 summarizes the efficacy of Exendin-4 on various organs and endpoints across different models.

**Table 3:** Summary table of the efficacy of Exendin-4 on various organs/endpoints across different models.

Modeling system	Species/cell	Exendin-4 treatment	Key findings (organ/endpoint)	Link to the FIRS pathology dimension	References
LPS-induced neuroinflammation models (in vitro/*in vivo*)	Rodents/Cell lines	Systemic administration	Decreased expression of IL-1β, TNF-α, and IL-6 proteins and mRNA in the hippocampus and cortex	Neuroinflammation	(30)
LPS-stimulated macrophage/NF-κB reporter system	cell line	Exogenous Exendin-4 treatment	Stabilizes IκBα, blocks the nuclear translocation of NF-κB p65, and inhibits the transcription of pro-inflammatory genes	Neuroinflammation	(31)
A study on metabolic reprogramming in Microglia (LPS model)	Primary microglia	Exendin-4 treatment	Induces the AMPK/SIRT1 pathway, promoting a shift in metabolism from glycolysis to oxidative phosphorylation, thereby favoring the M2 phenotype	Neuroinflammation/Immunometabolism	(32)
A model of systemic LPS exposure in newborn rats (simulating perinatal inflammation)	Newborn rats	Systemic treatment with Exendin-4	Decreased density of Iba1 + microglia in the brain; increased expression of Arg-1 and IL-10	Neuroinflammation, microglial phenotype	(33)
Cuprizone/Lysolecithin demyelination model	Rodents	Administration of Exendin-4	A decrease in OPC apoptosis; an increase in proliferation and in the number of MBP-positive mature oligodendrocytes; accelerated myelination; and improved motor coordination in the shuttle test.	OPC damage, myelin regeneration	(34)
Oligodendrocyte cell line/demyelinating model	Cells/Rodents	Treatment with Exendin-4	Activates the PI3K/Akt/mTOR pathway, promoting OPC survival and differentiation	OPC protection, myelin repair	(35)
Models related to cognitive function/BDNF research	Rodents	Exenatide (an Exendin-4 analogue)	Upregulates the BDNF–TrkB axis, supporting neuronal survival and axonal integrity, and promoting myelination	OPC-neuron interaction, myelination	(36)
CIH/Oxidative stress model	Rodents	Liraglutide (GLP-1RA)	Activates the Nrf2/HO-1 pathway, reduces oxidative stress, and protects oligodendrocytes from ROS-induced damage	Oxidative stress, oligodendrocyte protection	(37)
Diabetic encephalopathy model, MCAO cerebral ischemia model	Rats/mice	Systemic administration of Exendin-4	Decreased intracerebral Evans blue/IgG leakage; increased expression of ZO-1, occludin and claudin-5; decreased MMP-9 activity	BBB integrity	(38)
Epilepsy models, cerebral ischemia-reperfusion models	Rodents	Exendin-4 treatment	Alleviates NMDA receptor-mediated excitotoxicity; restores intracellular calcium homeostasis; reduces neuronal death	Excitotoxicity, calcium overload	(39)
Models of neurodegenerative diseases (autophagy research)	Cells/animals	Exendin-4	Induce moderate autophagy/mitochondrial autophagy to clear damaged mitochondria and toxic protein aggregates	Mitochondrial dysfunction	(40)
A palmitic acid-induced oxidative stress neuronal model	primary neurons	Exendin-4 treatment	Activates PGC-1α, promoting the biosynthesis of new mitochondria; improves ATP production; promotes neurite growth	Mitochondrial dysfunction, energy metabolism	(41)
Mouse model of Parkinson’s disease (gut microbiota research)	Mouse	Probiotics combined with GLP-1 pathway activation	Increases the abundance of SCFA-producing bacteria; indirectly protects the blood–brain barrier and modulates microglial function	The gut-brain axis (hypothesis)	(42)

### 5.1 Containing the neuroinflammatory storm and actively remodeling microglial phenotypes

In multiple, well-established *in vitro* and *in vivo* models of LPS-induced neuroinflammation, administration of Exendin-4 consistently and significantly reduces both the protein and mRNA expression levels of pivotal pro-inflammatory cytokines, including IL-1β, TNF-*α*, and IL-6, within critical brain regions such as the hippocampus and cerebral cortex (40). The upstream molecular mechanism governing this potent anti-inflammatory effect involves the suppression of the master regulator of inflammation, the nuclear factor kappa B (NF-κB) signaling pathway. Specifically, Exendin-4, through the activation of the GLP-1R/cAMP/PKA signaling cascade, stabilizes the NF-κB inhibitory protein, IκBα, preventing its phosphorylation and subsequent proteasomal degradation by the IKK complex. This action effectively blocks the nuclear translocation of the NF-κB p65 subunit and its binding to cognate DNA promoter sequences, thereby suppressing the transcriptional initiation of a wide array of pro-inflammatory genes (41). More notably, recent studies have begun to reveal that Exendin-4 can function as an “immune-metabolic tuner,” capable of influencing the fundamental polarization state of microglia by regulating their metabolic reprogramming. For instance, evidence suggests it may facilitate a shift in microglial metabolism away from a pro-inflammatory, glycolytic state (akin to the Warburg effect) and toward an anti-inflammatory, oxidative phosphorylation-based metabolic profile, potentially through modulation of the AMP-activated protein kinase (AMPK) and sirtuin 1 (SIRT1) pathways, thereby creating a metabolic milieu that favors the induction and sustenance of the protective M2 phenotype (42). Corroborating this, in a neonatal rat model of systemic LPS exposure, a paradigm that shares features with FIRS, Exendin-4 treatment reduced the overall activation density of Iba1-positive microglia and promoted their transition towards an M2 phenotype, as quantified by the up-regulation of Arg-1 and IL-10 expression, an immunomodulatory effect that was accompanied by significant improvements in behavioral outcomes (43). We therefore hypothesize that in the specific context of FIRS, Exendin-4 could function not as a conventional immunosuppressant, but as a sophisticated “immune recalibrator,” acting at both the transcriptional and metabolic levels to restore the imbalanced neuroimmune axis to a state of homeostasis, thereby creating a microenvironment that is conducive to brain tissue repair and regeneration.

### 5.2 Escorting oligodendrocyte development and actively promoting myelin regeneration

In established preclinical models of demyelination, such as those induced by cuprizone toxicity or lysolecithin application, Exendin-4 treatment has demonstrated striking and consistent pro-regenerative efficacy. It not only significantly reduces the rate of apoptosis among OPCs but also actively promotes their subsequent proliferation, successful differentiation into mature, myelin-basic-protein (MBP)-producing oligodendrocytes, and acceleration of the process of myelin sheath regeneration, as robustly corroborated by immunohistochemical staining for MBP and proteolipid protein (PLP) as well as by ultrastructural analysis via electron microscopy. These cellular and structural restorations translate functionally into measurable improvements in motor coordination and performance in behavioral tests, such as the rotarod test (44). The molecular mechanisms underpinning these restorative effects involve the synergistic activation of several key intracellular signaling pathways: (1) The PI3K/Akt/mTOR pathway, which is a central regulator of cell survival, proliferation, and protein synthesis, and its activation by Exendin-4 provides direct and potent support for OPC survival and their differentiation into mature OLs (30). (2) The CREB/BDNF pathway: Exendin-4-induced up-regulation of BDNF not only promotes the maturation of OPCs through autocrine actions on their endogenously expressed TrkB receptors but also, via paracrine mechanisms, provides crucial support for neuronal survival and axonal integrity, thereby ensuring a healthy axonal substrate necessary for effective ensheathment and myelination (45). (3) The Nrf2/HO-1 pathway: Exendin-4 also activates the central transcription factor Nrf2 (nuclear factor erythroid 2-related factor 2), leading to the upregulation of phase II detoxification and antioxidant enzymes, such as heme oxygenase-1 (HO-1), thereby protecting the highly vulnerable oligodendrocyte lineage from the pervasive oxidative damage that characterizes the FIRS inflammatory environment (46). We propose that this capacity for multi-pathway support is ideally, if not uniquely, suited to rescue the fragile oligodendrocyte lineage from the hostile, multifactorial assault it endures in the FIRS environment, offering a comprehensive protective strategy that supports the entire sequence of events from precursor survival and proliferation through to terminal differentiation and functional myelination, thereby maximizing the potential for salvaging white matter structure and function.

### 5.3 Reinforcement of blood–brain barrier integrity: an early intervention to sever the vicious cycle

In animal models of diabetic encephalopathy and in the widely used middle cerebral artery occlusion (MCAO) model of ischemic stroke, Exendin-4 treatment has been shown to significantly attenuate BBB disruption, as quantitatively assessed by reduced extravasation of Evans Blue dye and endogenous immunoglobulin G (IgG) into the brain parenchyma, and mechanistically attributed to the upregulation of key tight junction proteins, including ZO-1, occludin, and claudin-5 (47). The protective mechanism extends beyond its general anti-inflammatory effect (e.g., inhibition of TNF-*α*-induced junction disassembly) to include a more targeted modulation of vascular endothelial growth factor (VEGF) signaling—a known potent disruptor of BBB integrity—as well as the direct inhibition of the activity and expression of matrix metalloproteinase-9 (MMP-9), a zinc-dependent endopeptidase that plays a major role in the degradation of the extracellular matrix and the cleavage of tight junction proteins themselves (47). In the specific spatiotemporal context of FIRS, we posit that early intervention with Exendin-4 could act as a “reinforced dam” at the cerebral microvasculature to target a critical early node in the pathological cascade, thereby stabilizing the endothelial structure. This would prevent the initial “leak” of peripheral inflammatory mediators into the CNS compartment, thereby reducing the intensity and duration of neuroinflammation at its very source and effectively severing the vicious cycle of inflammation leading to BBB damage and subsequent amplification of inflammation before it becomes fully established.

### 5.4 Mitigation of excitotoxicity and enhancement of cellular resilience through autophagy and mitochondrial quality control

Exendin-4 has been demonstrated to attenuate glutamate-mediated excitotoxicity and associated mitochondrial dysfunction in models of epilepsy and cerebral ischemia–reperfusion injury, partly through modulation of NMDA receptor function and the stabilization of intracellular calcium homeostasis (48). A particularly compelling and evolutionarily conserved mechanism is its role as a potent inducer of macroautophagy (hereafter autophagy) (49). When moderately induced, the activation of this intracellular clearance pathway serves a vital cytoprotective function by selectively removing damaged organelles, particularly mitochondria (a process termed mitophagy), as well as aggregated or misfolded proteins. This not only eliminates potentially toxic cellular debris but also recycles components to yield energetic substrates, representing a crucial endogenous protective mechanism against various forms of cellular stress. By concurrently activating the PGC-1α signaling axis, Exendin-4 also promotes the biosynthesis of new, healthy mitochondria, thereby replacing the dysfunctional mitochondrial network and providing the injured neuron or oligodendrocyte with a new cohort of functional energy-producing organelles that are essential for recovery and sustained function (49). Thus, by targeting these final common pathways of cell death and dysfunction downstream of FIRS, Exendin-4 may comprehensively enhance the metabolic fitness and intrinsic resilience of neural cells through a dual strategy of “renewal”—promoting the generation of new components while efficiently clearing damaged components—enabling them to better withstand the multifaceted metabolic and oxidative stresses imposed by the inflammatory environment.

### 5.5 Emerging mechanisms: an intriguing role for the gut microbiota-brain axis?

An intriguing, albeit highly speculative, avenue of investigation involves the potential role of the gut-brain axis in mediating some of the systemic benefits of GLP-1RAs. A growing body of research suggests that GLP-1 receptor agonists, including Exendin-4, can exert systemic anti-inflammatory effects that may be partially mediated through favorable alterations in gut microbiota composition, for instance, by increasing the relative abundance of short-chain fatty acid (SCFA)-producing bacterial species (50). SCFAs themselves, such as butyrate, propionate, and acetate, have been independently shown to possess potent neuroprotective properties, including the ability to strengthen BBB function and modulate microglial activation. While this mechanism has been primarily studied in the context of peripheral inflammatory and metabolic diseases, it presents a provocative and testable hypothesis: could a portion of Exendin-4’s systemic anti-inflammatory and neuroprotective effects in the complex pathophysiology of FIRS be indirectly mediated through its action on the maternal and/or fetal gut microbiota? Exploring this potential “microbiota-brain axis” interaction represents a highly novel and worthwhile direction for future research, potentially uncovering a previously unappreciated dimension of its pleiotropic actions.

### 5.6 Developmental safety remains an important area of uncertainty

GLP-1 receptor agonists are known to affect embryonic growth in animal models, with studies reporting reduced fetal weight and increased skeletal abnormalities at suprapharmacological doses (51). Placental transfer of Exendin-4 is limited due to its peptide nature and molecular weight (~4 kDa), but recent evidence suggests detectable transplacental transfer in rodents, raising questions about fetal exposure in humans (52). Pharmacokinetically, the drug’s short half-life (2–4 h) necessitates twice-daily injections, and its clearance is primarily renal, limiting use in patients with moderate-to-severe renal impairment (53). Blood–brain barrier penetration remains a subject of debate: although some studies report peripheral administration leads to measurable CNS levels via circumventricular organs and saturable transport, the extent and functional significance remain debated, with most data derived from rodent models using supraphysiological doses (54). Potential adverse effects include nausea, vomiting, and pancreatitis, as well as rare cases of medullary thyroid carcinoma observed in rodents—a risk that contraindicates use in patients with MEN-2 (55). Finally, the limitations of current preclinical evidence are substantial: the majority of mechanistic studies use young, healthy male animals, lack long-term follow-up, and employ doses far exceeding clinically relevant doses, making translation to human safety and efficacy uncertain (56). Future investigations must address these gaps through rigorous, balanced experimental designs.

## 6 Challenges and innovative directions for future translational applications

Translating the compelling and multifaceted preclinical promise of Exendin-4 into a tangible clinical reality for infants affected by FIRS demands a realistic assessment of complex, intertwined challenges. Addressing these requires a commitment to correspondingly sophisticated and innovative solutions.

### 6.1 The paramount challenges of therapeutic timing, route, and developmental pharmacokinetics

The insidious and often clinically silent prenatal onset of FIRS presents a critical challenge in determining the optimal therapeutic window. The ideal scenario entails an intervention that is either administered prophylactically in very high-risk pregnancies or initiated very early within the evolving injury cascade—specifically within a putative “therapeutic time window”—prior to the consolidation of irreversible damage.

Proposed Solution Strategy: Future efforts should prioritize developing and validating AI-driven predictive models that integrate multi-component biomarker panels (e.g., specific cytokine combinations, ratios of cytokines to neuroproteins, circulating non-coding RNAs) with advanced fetal imaging metrics (e.g., from diffusion tensor MRI) to facilitate ultra-early antenatal risk stratification. Such an approach would establish a critical therapeutic window for targeted maternal interventions.

Addressing the Critical Challenge of Placental Pharmacokinetics: The extent to which GLP-1RAs, including Exendin-4, cross the human placental barrier remains poorly characterized and represents a significant knowledge gap (57, 58). The extent of transfer likely depends on factors such as molecular size, lipophilicity, and interactions with placentally expressed DPP-4. Therefore, systematic and rigorous studies using ex vivo human placental perfusion models, complemented by comprehensive pharmacokinetic and safety studies in pregnant animal models, represent an urgent and critical research priority to determine whether effective fetal drug levels can be achieved via maternal administration. Furthermore, the insidious prenatal onset of FIRS necessitates either prophylactic maternal administration in high-risk pregnancies or immediate postnatal intervention. However, maternal dosing is critically constrained by the limited understanding of Exendin-4 placental transfer. According to the U. S. FDA-approved prescribing information for exenatide, exenatide crosses the placenta in animal models at clinically relevant exposures, leading to reduced fetal weight and skeletal abnormalities at doses 0.5–6 times the human exposure. This highlights the urgent need for systematic pharmacokinetic studies in pregnant animal models to determine whether both safe and therapeutic fetal concentrations can be achieved via maternal administration. Neonatal administration bypasses the placental barrier but may be complicated by a narrowed therapeutic window and immature drug metabolism. Rigorous developmental toxicology studies in large animal models are essential to assess impacts on fetal growth and organogenesis, while long-term neurobehavioral follow-up into adolescence is imperative to rule out subtle cognitive or emotional deficits. Furthermore, direct comparative preclinical studies with longer-acting, more potent GLP-1 receptor agonists (liraglutide, semaglutide) and dual-target agents are warranted, as these compounds may offer superior neuroprotective efficacy or improved safety profiles in FIRS. Ultimately, a personalized, biomarker-driven approach will be essential to optimize treatment timing, dosing, and agent selection for individual patients (59).

### 6.2 Rigorous developmental toxicological and long-term neurobehavioral safety assessments

The developing brain is exquisitely sensitive to pharmacological intervention, and the potential for unintended, subtle, long-term neurodevelopmental consequences must be addressed with the utmost rigor. Beyond standard toxicology, extensive developmental toxicology studies conducted in translationally relevant large animal models (e.g., the preterm fetal sheep model of FIRS) are essential to assess potential impacts on fetal growth patterns, organogenesis, and, most critically, complex brain development (60). Furthermore, long-term neurobehavioral follow-up into adolescence and adulthood in these animal models, employing sophisticated cognitive testing batteries, detailed social and emotional behavioral analyses, and advanced neuroimaging, is imperative to ensure with a high degree of confidence that the treatment does not exert any deleterious, albeit subtle, effects on higher-order brain functions such as learning, memory, executive function, and emotional regulation.

### 6.3 The imperative of personalized medicine: overcoming heterogeneity with biomarkers

FIRS is not a monolithic entity but exhibits profound etiological and pathophysiological heterogeneity. A simplistic uniform therapeutic strategy is therefore likely to result in limited efficacy and potential failure. Positioning Exendin-4 within the broader landscape of investigational neuroprotective strategies for neonatal brain injury significantly enhances the translational perspective. Several promising interventions are under active investigation, each with distinct mechanisms and limitations. Erythropoietin (EPO) offers pleiotropic anti-apoptotic and anti-inflammatory effects (61), yet its optimal dosing window for FIRS remains undefined. Melatonin is a potent antioxidant but is limited by poor bioavailability in neonates. Magnesium sulfate targets excitotoxicity and is established for maternal neuroprotection in preterm birth, but it may insufficiently address the sustained inflammatory cascades of FIRS (62). Mesenchymal stem cell (MSC) therapy holds potential for cell-based immunomodulation, but it faces significant hurdles in scalability, timing, and long-term safety evaluation (63). Finally, therapeutic hypothermia, the standard of care for hypoxic–ischemic encephalopathy (HIE), is less applicable to FIRS, where inflammation-driven injury often initiates prenatally and may not respond to postnatal cooling alone (64). Given these limitations, GLP-1-based strategies—particularly next-generation agonists like semaglutide and tirzepatide—offer a unique advantage by directly targeting neuroinflammation and metabolic stress through well-characterized signaling pathways. Future clinical trial designs must move beyond population-level analysis and incorporate dynamic biomarker panels to map individual injury-repair trajectories. Furthermore, we propose that head-to-head preclinical comparisons of Exendin-4 against EPO, melatonin, magnesium, MSC therapy, and hypothermia in validated FIRS models are critically needed. Such comparisons, combined with explorations of rational combination therapies, will be essential to identify the most effective and safest intervention for advancing the field of precision neonatology.

Proposed Solution Strategy: Future clinical trial designs must move beyond population-level analysis and incorporate dynamic, responsive biomarker panels. For example, the serial measurement of biomarkers such as GFAP (reflecting astrocyte injury), neurofilament light chain (NF-L, indicating axonal injury), Tau protein, and multiplex cytokine profiles in neonatal serum or plasma would enable the mapping of an individual infant’s unique “neurological injury-repair trajectory” and the objective assessment of their specific biological response to Exendin-4 treatment. Ultimately, this data-driven approach is the cornerstone of achieving precision neonatology (65).

### 6.4 Looking beyond Exendin-4: the promise of next-generation agonists and rational combination therapies

The landscape of GLP-1-based pharmacology is evolving rapidly, offering opportunities to utilize increasingly potent or targeted agents.

Next,-Generation GLP-1RAs: Compounds such as liraglutide and notably semaglutide offer significantly extended plasma half-lives, permitting once-daily or even once-weekly dosing, and may possess greater receptor activation potency, potentially leading to improved neuroprotective outcomes (66).

Dual- and Multi-Target Agonists: A New Therapeutic Paradigm. This class of agents represents a significant paradigm shift in pharmacotherapy. Agents like the GLP-1/GIP dual agonist tirzepatide have demonstrated remarkable efficacy in metabolic diseases; given the widespread distribution of the GIP receptor in the brain, its co-activation may confer additional, complementary neuroprotective benefits (67). Similarly, GLP-1/glucagon dual agonists, in which glucagon receptor activation may promote beneficial energy expenditure and neural activity, hold significant potential. We propose that a critical and forward-looking research direction involves the head-to-head preclinical evaluation of these novel, next-generation agonists against Exendin-4 in well-validated FIRS models, to determine whether simultaneous activation of multiple, complementary signaling pathways can achieve a more comprehensive, robust, and efficient suppression of the neuroinflammatory network, metabolic stress, and resultant cellular damage.

## 7 Conclusion and future perspectives

Repositioning Exendin-4 from a promising neuroprotective agent in preclinical models to a clinically validated, safe, and effective therapy for FIRS is an ambitious yet potentially transformative endeavor. Its success will demand an interdisciplinary collaboration that bridges fundamental neuroscience, medicinal chemistry, obstetric and neonatal pharmacology, advanced bioanalytics, and clinical trial design. By embracing a strategic and nuanced approach to precision neuroprotection—one that leverages innovative CNS-targeted delivery systems, biomarker-guided personalization, and the rational exploration of next-generation multi-target agents—we can aim to establish a powerful and timely “therapeutic umbrella” for the most vulnerable of patients, the developing fetus and newborn. The ultimate goal is to safeguard their neurological future, mitigate the burden of neurodevelopmental disability, and fundamentally improve their lifelong quality of life, an objective that represents a paramount objective in medical science.
